# The influence of depressive and manic symptoms on suicidal ideation in mixed mood states

**DOI:** 10.1186/s40345-025-00390-x

**Published:** 2025-06-14

**Authors:** Monica Macellaro, Rita Cafaro, Carlton Max Kelly, Michael J. Ostacher, Bernardo Dell’Osso, Jihun Lyu, Mark A. Frye, Ralph W. Kupka, Susan L. McElroy, Willem A. Nolen, Paul E. Jr Keck, Robert M. Post, Heinz Grunze, Trisha Suppes

**Affiliations:** 1 Department of Biomedical and Clinical Sciences “Luigi Sacco”, Department of Psychiatry, University of Milan, Milan, Italy; 2 Department of Veterans Affairs Palo Alto Health Care System, 3801 Miranda Ave. 151T, Palo Alto, CA 94304 USA; 3 Department of Psychiatry and Behavioral Sciences, Stanford University School of Medicine, Stanford University, Stanford, CA USA; 4 Department of Health Sciences, “Aldo Ravelli” Center for Neurotechnology and Brain Therapeutic, University of Milan, Milan, Italy; 5 Department of Clinical Psychology, Palo Alto University, Palo Alto, CA USA; 6 Department of Psychiatry & Psychology, Mayo Clinic, Rochester, MN USA; 7 Department of Psychiatry, Amsterdam University Medical Center, Amsterdam, Netherlands; 8 Lindner Center of HOPE, University of Cincinnati, Mason, Cincinnati, OH, OH USA; 9 Department of Psychiatry, University Medical Center Groningen, University of Groningen, Groningen, Netherlands; 10 Clinical Professor of Psychiatry, George Washington School of Medicine, Washington, DC USA; 11 Department of Psychiatry, Psychiatrie Schwäbisch Hall &, Paracelsus Medical University, Nuremberg, Germany

**Keywords:** Bipolar disorder, Mania, Depression, Mixed States, Mixed features, Mixity, Suicidal ideation

## Abstract

**Background:**

While bipolar disorder is strongly linked to an increased risk of suicide, recent evidence has challenged the assumption that mixed symptoms play a distinct role in suicidal ideation beyond depressive severity. This study examines how depressive, hypo/manic, and mixed features influence suicidal ideation in individuals with bipolar disorder. Data from 903 participants in the Stanley Foundation Bipolar Network (1995–2002) were analyzed to assess associations between mood states, classified by the Inventory of Depressive Symptomatology–Clinician-Rated (IDS-C) and the Young Mania Rating Scale (YMRS), and suicidal ideation, measured using IDS-C item 18, using generalized estimating equations.

**Results:**

Depressive symptoms were strongly associated with suicidal ideation (OR = 21.98, 95% CI: 15.31–31.54). Moderate hypo/manic symptoms also conferred risk (OR = 3.11, 95% CI: 1.51–6.49), and milder hypo/mania showed a weaker but significant association (OR = 1.74, 95% CI: 1.05–2.89). The highest suicidal ideation was observed in individuals with hypo/mania featuring mixed symptoms (OR = 29.43), exceeding that of depression or depression with mixed features (OR = 21.98). However, findings diverged based on modeling approach: in continuous predictor models, SI was driven solely by depressive symptom severity, with no significant association observed for hypo/mania or its interaction with depression. In contrast, when mood states were categorized using clinically meaningful thresholds, hypo/mania with mixed features emerged as a distinct contributor to suicidal ideation risk.

**Conclusion:**

These findings underscore the need for integrating both dimensional and categorical approaches to mood state classification in research on suicidality in bipolar disorder.

**Supplementary Information:**

The online version contains supplementary material available at 10.1186/s40345-025-00390-x.

## Background

Individuals with bipolar disorder (BD) face a markedly elevated risk of suicide, with estimates suggesting a 20–30-fold increase compared to the general population [1]. The International Society for Bipolar Disorder (ISBD) Task Force on Suicide in BD reported an annual suicide rate of 164 per 100,000 individuals [2]. While mixed symptoms have long been considered a key risk factor for suicidality in BD, recent evidence suggests that their association with suicidal ideation (SI) may primarily reflect the severity of underlying depressive symptoms rather than an independent effect.

Mixed states are characterized by the co-occurrence of manic and depressive symptoms and are common among individuals with BD [3–9]. Historically, the Diagnostic and Statistical Manual of Mental Disorders, Fourth Edition (DSM-4) defined mixed episodes narrowly, requiring patients to simultaneously meet full diagnostic criteria for both a major depressive episode and a manic episode [10]. This definition was restricted to individuals with bipolar I disorder (BD I) and excluded presentations with subthreshold symptoms.

With the publication of the DSM-5 and the International Classification of Diseases, 11th Revision **(**ICD-11), the classification of mixed episodes was revised. DSM-5 introduced the “with mixed features” specifier, allowing for the recognition of mixed symptoms even if full criteria for both mood states are not met [11]. Unlike the DSM-4 framework, this specifier accounts for subthreshold symptoms, potentially increasing the identification of individuals with mixed states. ICD-11, in contrast, classifies mixed episodes as a distinct entity, emphasizing the clinical prominence of mixed mood states rather than a strict symptom threshold [12]. 

There is some evidence indicating that mixed states may heighten suicide risk. For instance, individuals experiencing mania with mixed features report higher levels of suicidal ideation (SI) compared to those with pure mania [13–15]. Prior studies have found that mixed states were associated with a greater risk of SI, attempts, and completed suicide compared to pure hypo/mania [16, 17]. Supporting this further, a 5-year prospective study and found that suicide attempts occurred most frequently during mixed states, with an incidence rate over 120 times higher than during euthymia [18]. However, it remains unclear whether mixed states confer a higher risk of suicide or if this is a higher risk due to more severe depression during a mixed state compared to pure depression [16, 19, 20].

Recent research has raised key questions on this issue of mixed states and suicidal ideation or attempts. Some studies suggest that mixed states do not independently increase SI or behavior beyond the effects of depressive symptom severity [21–23]. For example, Persons and colleagues found that individuals with BD I and a history of mixed states were more likely to exhibit suicidal behavior when depressed compared to those without such a history. However, 71% of this excess risk was attributed to time spent in depressive episodes [23]. Similarly, Fiedorowicz et al. reported that SI and behavior were strongly associated with depressive symptoms, whereas manic or mixed symptoms, including hypo/mania with mixed features, did not show a significant independent relationship [21, 22]. 

These findings challenge the notion that mixed symptoms confer unique suicide risk and instead suggest that depressive severity is the primary driver of SI in BD. Whether mixed mood states confer additional risk for SI beyond depression alone remains a subject of ongoing debate.

Clarifying the relationship between mixed symptoms and SI is essential for improving risk assessment and informing targeted intervention strategies. This study aims to examine the association between SI and depressive, hypomanic/manic, and mixed mood states over time, while also exploring whether gender or bipolar subtype moderates this relationship. By comparing both categorical and continuous models, we seek to determine whether mixed symptoms independently predict SI or if their effects are accounted for by depressive severity only.

## Methods

### Study design and procedures

The methods and procedures for the Stanley Foundation Bipolar Treatment Outcome Network (SFBN) study (1995–2002) have been previously described in detail [24, 25]. All participants provided written informed consent, approved by the respective local Institutional Review Boards (IRBs), prior to study enrollment. Study details are described in brief below.

### Participants

A total of 935 adults were recruited from four U.S. and three European sites for this naturalistic, longitudinal study, which involved the prospective assessment of clinical mood states. All participants were diagnosed using the Structured Clinical Interview from the DSM-4 [10]. Patients were evaluated at least monthly, with treatment adjustments made as clinically indicated. Patients met DSM-4 criteria for bipolar I disorder (BD-I), bipolar II disorder (BD-II), bipolar disorder not otherwise specified (BD-NOS), or schizoaffective disorder–bipolar type. Patient characteristics stratified by bipolar disorder subtype are presented in Table 1. An interrater reliability was maintained between clinical sites (κ values: YMRS, 0.7 and IDS-C, 0.85) [4]. 

**Table 1:** Distribution of bipolar subtypes, gender, and age among participants with and without suicidal ideation across all visits

	All subjects	Subjects with no visits with suicidal ideation	Subjects with one or more visits with suicidal ideation
	(N = 903 [100%])	(N = 352 [39.0%])	(N = 551 [61.0%])
**Bipolar Type**			
BDI	677 (75.0%)	260 (73.9%)	417 (75.7%)
BDII	186 (20.6%)	73 (20.7%)	113 (20.5%)
BD NOS	18 (2.0%)	10 (3.1%)	8 (1.5%)
SD– Bipolar type	22 (2.4%)	9 (2.8%)	13 (2.4%)
**Gender**			
Female	505 (55.9%)	207 (58.8%)	298 (54.1%)
Male	398 (44.1%)	145 (41.2%)	253 (45.9%)
**Age**	40.98 ± 11.53	40.63 ± 11.87	41.41 ± 11.32

Suicidal Ideation is here defined as a score ≥ 1 at the IDS-C - item 18; BDI: Bipolar Disorder, type I; BDII: Bipolar Disorder, type II; BD NOS: Bipolar Disorder, Not Otherwise Specified; SD– Bipolar type: Schizoaffective Disorder– Bipolar type

Individuals who participated in defined, open-label, or double-blind clinical trials were excluded from the cohort [24, 26]. The current analyses focuses on patients who completed at least one visit with concurrent assessments of manic and depressive symptoms using the Young Mania Rating Scale (YMRS) [27] and the Inventory of Depressive Symptomatology–Clinician-Rated (IDS-C) [28, 29]. 

### Mood state assessment

Mood states were classified using scores from the YMRS and the IDS-C, both of which are clinician-administered and have demonstrated strong reliability and validity [27–29]. These measures were completed during study visits, with symptoms assessed on the day of visit and for the preceding 3 and 7 days for YMRS and IDS-C, respectively.

To identify mood states, we applied symptom-based cut-offs derived from prior work which used YMRS and IDS-C thresholds in a naturalistic study of SFBN data (see Table 2) [4, 30]. These mixed state definitions are not formal diagnostic criteria, but rather dimensional, symptom-based constructs operationalized for the purposes of this study. Depressive symptoms were defined as an IDS-C R score of 15 or higher, indicating at least mild depression. Depression with mixed features was defined as the co-occurrence of depressive symptoms (IDS-C *R* ≥ 15) alongside mild hypo/manic symptoms, as indicated by YMRS scores between 3 and 11. Hypo/mania with mixed features was defined as the presence of depressive symptoms (IDS-C *R* ≥ 15) with moderate hypo/manic symptoms, characterized by YMRS scores of 12 or greater.

**Table 2:** Definitions of mood States by symptom scales

	IDS-C	YMRS
**Symptom category**		
Depressive symptoms	≥ 15	-
Mild hypomanic symptoms	-	> 2 and < 12
Moderate hypo/manic symptoms	-	≥ 12
***Mood state***		
Pure depression	≥ 15	≤ 2
Depression with mixed features	≥ 15	> 2 and < 12
Pure hypo/mania	< 15	≥ 12
Hypo/mania with mixed features	≥ 15	≥ 12
Euthymia	< 15	< 12

IDS-C: Inventory of Depressive Symptomatology-Clinician-Rated Version (assessed day of and prior 7 days); YMRS: Young Mania Rating Scale (assessed day of and prior 3 days)

For depression symptom categorization, we used the full IDS-C score; however, for statistical analyses, we utilized a modified version of the IDS-C (IDS-C R) in which item 18 (suicidal ideation) was removed to avoid circularity, ensuring that the predictor (depression severity) does not include variance from the outcome variable. The IDS-C and IDS-C R scores were highly correlated (*r* = 0.99, *p* < 0.0001).

The YMRS and IDS-C share several items that assess overlapping symptoms, including sleep disturbances, irritability, and psychomotor agitation. To determine whether the observed associations were driven by these overlapping symptoms rather than distinct mood state effects, we conducted a sensitivity analysis in which these items were removed. Specifically, we excluded items 4 (Sleep) and 5 (Irritability) from the YMRS, and items 1–3 (Insomnia), 6 (Irritability), and 24 (Psychomotor agitation) from the IDS-C in subsequent analyses. This allowed us to isolate the relationship of interest from potential artifact.

### Suicidal ideation assessment

SI was assessed using item 18 of the IDS-C, which evaluates the presence and severity of suicidal thoughts. Item 18 is scored as follows: 0 indicates no thoughts of suicide or death; 1 indicates a sense of emptiness or the belief that life is not worth living; 2 indicates suicidal thoughts occurring several times per week for several minutes; and 3 indicates suicidal thoughts occurring daily, with or without a plan or past suicide attempt. For our analyses, we dichotomized suicidal ideation to IDS-C item 18 = 0 and IDS-C item 18 ≥ 1.

### Statistical analysis

Statistical analyses were conducted using SPSS version 28 (IBM Corp., Armonk, NY, USA). Generalized estimating equations (GEE) were used to examine the association between mood symptoms—specifically depressive, hypo/manic, and their co-occurrence—and SI assessing both the strength and direction of these relationships. The primary analyses utilized categorical models, with mood symptoms categorized based on predefined thresholds that capture distinct mood presentations.

The GEE models were specified using binary logistic regression with a robust covariance estimator and an independent working correlation matrix to account for repeated observations within individuals. The dependent variable in all models was the IDS-C item 18 score, which reflects the presence and severity of SI. The primary predictor variables were the categorical indicators of depressive, moderate, and mild hypo/manic symptoms. An interaction term was included to capture the potential synergistic effect of co-occurring depressive and hypo/manic symptoms.

The interpretation of the GEE model coefficients followed conventional guidelines for binary logistic regression. A positive β-value indicates an increased likelihood of suicidal ideation as symptom severity increases, whereas a negative β-value suggests a decreased likelihood. Odds ratios (OR) were derived from the β-coefficients using the standard transformation OR = e^(β)^ providing a measure of odds for SI based on mood symptom severity. A statistically significant interaction term indicates that the relationship between mood symptoms SI differs from the additive contributions of depressive and hypo/manic symptoms. Age and study week were included as linear covariates in all models to control for potential time- and age-related effects. Additional covariates included gender, both as a main effect and in interaction with mood symptoms, and bipolar subtype.

To assess the combined effect of depressive and hypo/manic symptoms, we calculated combined ORs by exponentiating the sum of the relevant β-coefficients (e.g., OR_combined_ = exp^(β_depression + β_hypomania + β_interaction)^). This allowed us to quantify how co-occurring depressive and hypo/manic symptoms modify SI beyond their individual contributions. For combined OR calculations, only statistically significant β-values were included to ensure that the resulting estimate reflected meaningful associations. If a β-coefficient was not significant (*p* > 0.05), its corresponding OR was not included in the combined calculation, as its contribution to the overall effect was uncertain. Similarly, if the revised scales (e.g., excluding overlapping items) altered statistical significance, the corresponding OR was excluded, ensuring that the final combined OR was based only on robust, validated effects.

In addition to the primary categorical models, secondary analyses were conducted using continuous YMRS and IDS-C R scores as predictor variables to replicate methodology from prior research [21–23, 31]. The continuous models followed the same GEE specifications as the categorical models; however, instead of dichotomizing symptoms, mood symptoms were modeled as continuous variables to capture variability in symptom severity without the use of predefined clinical categories. The interaction between continuous depressive and hypo/manic symptom scores was included to identify potential nonlinear relationships and mixed symptom presentations.

## Results

### Patients’ demographics and duration of follow-up

Out of the original cohort of 935 adults, 903 patients had completed at least one visit with both YMRS and IDS-C assessments (mean age = 40.98 ± 11.53, 55.9% female; Table 1). Across 14,213 visits, patients had a median of 12 visits (mean = 16.6 ± 14.2) over a median follow-up of 15 months (mean = 22.7 ± 22.9 months). Suicidal ideation scores ≥ 1 were recorded in 17.2% (2,449 visits), scores ≥ 2 in 6.9% (977 visits), and scores = 3 in 1.1% (160 visits).

### Visits and mood states

**Fig. 1:**
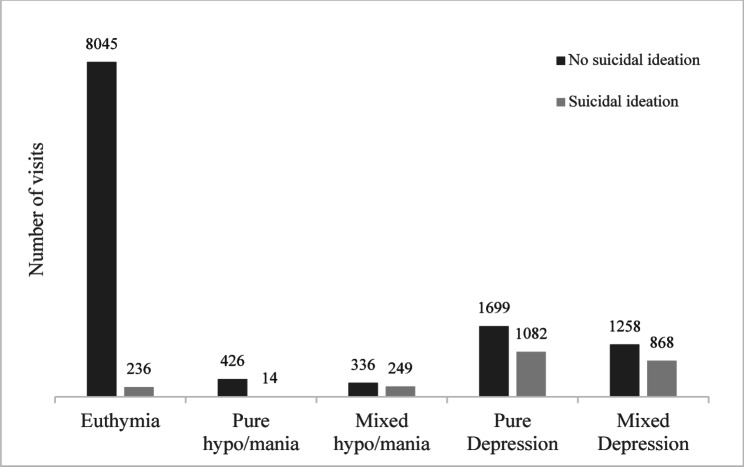
Number of visits in which suicidal ideation was detected by mood state Euthymia is defined as an IDS-C score < 15 and a YMRS score < 12; pure hypo/mania is defined as an IDS-C score < 15 and a YMRS score ≥ 12; hypo/mania with mixed features is defined as an IDS-C score ≥ 15 while YMRS score ≥ 12; pure depression is defined as an IDS-C score ≥ 15 while YMRS score ≤ 2; depression with mixed features is defined as a concomitant IDS-C score ≥ 15 and a YMRS score > 2 and < 12. Suicidal ideation is here defined as a score ≥ 1 at the IDS-C– item 18

Among 14,213 visits, the majority (*N* = 8,281 [58.3%]) occurred during a euthymic phase (Fig. 1). Depressive symptoms (IDS-C score ≥ 15) were present in 4,907 visits (34.5%). Of these, 2,781 (19.6%) were characterized as pure depression and 2,126 (14.9%) as depression with mixed features (> 2 YMRS < 12 and IDS-C ≥ 15). Furthermore, 1,025 visits (7.2%) reported hypo/manic symptoms, of which 440 visits (3.1%) reached scores for pure hypo/mania (YMRS ≥ 12), while 585 visits (4.1%) were characterized as hypo/mania with mixed features (IDSC-C ≥ 15 and YMRS ≥ 12).

### Visits and suicidal ideation

An IDS-C item 18 score of ≥ 1 was observed in 2,449 visits (17.2%), with 977 visits (6.9%) showing a score of ≥ 2 and 160 visits (1.1%) showing a score of 3. The distribution of SI across different mood states is shown in Fig. 1.

### Relationship between suicidal ideation and categorical indicators of mood states

Results from the GEE models using categorical variables for depressive symptoms, hypo/manic symptoms, and their interaction are summarized in Table 3. Depressive symptoms were strongly related to SI (β = 3.09, 95% CI: 2.73–3.45). Moderate hypo/manic symptoms had a smaller but significant association with SI (β = 1.135, 95% CI 0.41–1.87) and mild hypo/mania demonstrated a modest contribution (β = 0.552, 95% CI: 0.05–1.06). There was a significant interaction effect between moderate hypo/mania and depression (β = -0.843, 95% CI: -1.59– -0.10).

**Table 3:** Associations of depressive and hypomanic symptoms on suicidal ideation

	Suicidal ideation ≥ 1		
	β	95% CI	*P* value ^a^
Depressive symptoms	3.09	2.73–3.45	0.000
Moderate hypo/manic symptoms	1.135	0.41–1.87	0.002
Mild hypo/manic symptoms	0.552	0.05–1.06	0.032
Interaction between depressive and mild hypo/mania symptoms	-0.43	-0.95–0.91	0.105
Interaction between depressive and moderate hypo/mania symptoms	-0.843	-1.59– -0.1	0.026

CI: confidence intervals

See eTable 2 for full details, including additional predictors and reference categories

^a^ GEE models used Bonferroni correction to adjust for multiple comparisons

When using the revised YMRS scale with overlapping items removed, the findings for hypo/mania with mixed features remained consistent, with significant contributions from depression, hypo/mania, and their interaction. However, the previously observed association between mild hypo/mania and suicidal ideation was no longer significant (β = 0.308, 95% CI: -0.14 to 0.76) and was therefore excluded from the combined odds for depression with mixed features (see eTable 1 in the Supplement).

Table 4 presents the odds of SI for each mood category relative to euthymia, calculated using beta coefficients from the GEE models. Depression was associated with significantly higher odds of SI (OR = 21.98, 95% CI: 15.31–31.54), while moderate and mild hypo/manic symptoms were associated with smaller but significant odds (OR = 3.11, 95% CI: 1.51–6.49; OR = 1.74, 95% CI: 1.05–2.89).

**Table 4:** Effects of pure and mixed mood States on suicidal ideation relative to euthymia

Symptom Category	Odds Ratio	95% CI
Depression	21.98	15.31–31.54
Moderate hypo/mania	3.11	1.51–6.49
Mild hypo/mania	1.74	1.05–2.89
**Mixed Mood State**		
Depression with mixed features	21.98	
Hypo/mania with mixed features	29.43	

CI: confidence intervals

Euthymia is defined as an IDS-C score < 15 and a YMRS score < 12; hypo/mania with mixed features is defined as an IDS-C score ≥ 15 while YMRS score ≥ 12; pure depression is defined as an IDS-C score ≥ 15 while YMRS score ≤ 2; depression with mixed features is defined as a concomitant IDS-C score ≥ 15 and a YMRS score > 2 and < 12. Suicidal ideation is here defined as a score ≥ 1 at the IDS-C– item 18. The odds of suicidal ideation for each symptom category were calculated using GEE. Beta coefficients were excluded from the combined odds ratios for mixed mood states if they were not statistically significant, demonstrated instability across revised scales, or showed unreliable variance estimates. Confidence intervals for the combined odds ratios were not calculated due to the complexity of estimating the variance of interaction terms, which depends on the model’s variance-covariance structure and cannot be reliably derived with available data

For mixed mood states, depression with mixed features had the same odds of SI as pure depression (OR = 21.98), as its calculation included only the depressive symptom component, with mild hypomania and its interaction with depression excluded. Hypo/mania with mixed features had the highest odds of SI (OR = 29.43), calculated from the individual effects of mania, depression, and their interaction term. Confidence intervals for the mixed mood states were not reported due to variance instability.

### Relationship between suicidal ideation and other predictors

Gender significantly moderated the relationship between mood state and SI, with a protective effect for males in pure hypo/mania (β = -1.451, 95% CI: -2.58– -0.32) while age and bipolar subtype did not demonstrate any significant relationship (eTable 2 in supplement).

### Relationship between suicidal ideation and continuous IDS-C R and YMRS scores

A secondary GEE model was built using continuous variables as predictor variables. Results are shown in eTable 3 in the Supplement. In the continuous models, depressive symptom severity was significantly associated with increased suicidal ideation (β = 0.133, *p* < 0.001), while hypo/manic symptoms (β = 0.022, *p* = 0.115) and their interaction (β = -0.001, *p* = 0.252) were not significant predictors.

## Discussion

In this large well-characterized sample of patients with bipolar disorder 60.7% of participants had at least one visit where broadly defined SI was present (IDS-C item 18 score ≥ 1). When mood states were categorized by degree of severity, hypo/mania with mixed features emerged as a distinct risk factor for SI, suggesting that threshold effects captured by categorical models are not captured in previous continuous analyses. Using categorical mood states as predictors, hypo/mania with mixed features had the highest observed odds of SI (OR = 29.43), whereas depression with mixed features had the same odds as pure depression (OR = 21.98). The increased risk in hypo/mania with mixed features was driven by significant contributions from mania, depression, and their interaction term. In contrast, for depression with mixed features, the odds of SI were based solely on the depressive symptom contribution, as mild hypo/mania and its interaction with depression were not statistically significant or lacked robustness in sensitivity analyses, leading to their exclusion from the final calculation.

In order to categorize and differentiate between mixed states, as used in earlier studies with this population, a lower YMRS cutoff (3–11) was used to define depression with mixed features, capturing concurrent hypo/manic symptoms, while a higher YMRS threshold (12 or greater) identified hypo/mania with mixed features. In the primary analyses using the full scales, both moderate and mild hypo/mania were significantly associated with an increased likelihood of SI, but in the sensitivity analyses where overlapping items between the YMRS and IDS-C were removed, the association between mild hypo/mania and SI was no longer significant, suggesting that SI in this context is driven by depressive symptoms alone. This pattern indicates that overlapping symptoms, such as irritability and sleep disturbances, may have contributed to the observed relationship between mild hypo/mania and SI when YMRS scores fell between 3 and 11.

To replicate prior studies, we tested whether the association between mixed mood states and SI was primarily driven by the presence of depressive symptoms alone, or by the combination of depressive and hypomanic symptoms. Using continuous mood symptom scores, we found that depressive symptoms accounted for the observed association with SI, while hypomanic symptoms and their interaction with depression did not significantly contribute. These findings align with previous research indicating that SI in mixed states may largely reflect the influence of depressive symptoms.

A summary of earlier work in this area includes three different populations of patients with bipolar disorder. Firstly, data from the National Network of Depression Centers (NNDC) Mood Outcomes Program showed that depressive symptoms assessed with the Patient Health Questionnaire (PHQ-8) were the strongest predictor of SI, while manic symptoms measured with the Altman Self-Rating Mania Scale (ASRM) were not associated with increased suicide risk in bipolar disorder and were inversely associated with SI in major depression [21]. An additional analysis of NNDC data found that neither manic nor anxiety symptoms independently contributed to SI [31]. Similarly, in an analysis of the NNDC Clinical Care Registry found that depressive symptoms assessed with the PHQ-9 strongly predicted SI and behavior, while manic symptoms and mixed states—whether modeled continuously or categorically—were not significantly associated with suicide risk [22]. These findings suggest that across different assessment methods, the relationship between depressive symptoms and SI remains consistent when mood symptoms are treated as continuous variables.

Lastly, prospective data from the NIMH Collaborative Depression Study followed 429 individuals with bipolar disorder over an average of 18 years and similarly supports this pattern. Using the LIFE Psychiatric Status Rating (PSR) scale to characterize mood states, the study identified depression as a high-risk state for suicidal behavior, while mixed states did not confer additional risk beyond the depressive component [23]. A limitation of the NIMH study is that while prospective mood symptoms may only have been queried every 6 or 12 months making complete mixed mood descriptions limited.

This body of work suggests that threshold effects captured by categorical models are not adequately reflected in continuous analyses. The categorical models in our study revealed an increased odds of SI associated with moderate hypo/mania with mixed features, which diverges from findings in studies using continuous analyses.

Much of the prior research primarily relied on self-reported mood assessments such as PHQ-8, PHQ-9, or ASRM. In contrast, our study used monthly clinician-rated IDS-C and YMRS scores and predefined categorical thresholds for mood states. It should be noted the study of the NNDC registry analysis used the Columbia-Suicide Severity Rating Scale (C-SSRS) to assess SI and behavior, while our study relied on item 18 of the IDS-C. It is also important to note that our definition of mixed features differs from those used in the NNDC and NIMH studies due to differences in measurement tools and threshold scores required to define mood states. These methodological variations may contribute to the observed discrepancies in categorical findings.

The divergence between the categorical and continuous models raises important considerations regarding how mixed symptoms are best characterized in relation to SI risk. One possible explanation is that categorical models, which define mood states using clinical thresholds, may better capture the episodic nature of bipolar disorder and its relevance to SI, whereas continuous measures assume a linear relationship that may not fully account for dynamic symptom interactions. The use of categorical models may allow for the identification of clinically meaningful subgroups at increased risk, while continuous models may fail to capture nonlinear relationships between symptom severity and SI risk. These differing analytic approaches highlight the complexity of assessing mood states in bipolar disorder and suggest that future studies should explore the potential advantages and limitations of each method.

Prior research also found that women are more likely than men to meet DSM-5 criteria for mixed hypomania or mania [30]. Women also experience depressive symptoms more frequently during hypomania, whereas men’s depressive symptoms are primarily characterized by irritability and agitation [4]. While gender did not significantly impact suicidal ideation in mixed states in our study, it did interact with pure hypomania, reducing SI risk in men. This is consistent with prior research indicating that manic symptoms increase SI risk in women but not in men, as observed in NNDC findings [21, 22]. 

The study has significant limitations which limit the interpretability of the results. The presence of SI was assessed only through item 18 of the IDS-C, therefore it may not be generalized to other definitions of suicide. As such, the conclusions drawn from this analysis are more relevant to the odds of SI than to the odds of suicide attempt or completed suicide. The GEE models used in our analyses assume independence of observations across different study sites, which may not fully capture site-specific variations and could impact the robustness of our findings. The use of mood symptom rating scales involved empirical cut-offs for symptoms, and it was not possible to differentiate whether symptoms present at visits represented new or persistent mood symptoms, thus limiting our interpretation of the longitudinal trajectories and the casual effect of mixed symptoms. The mixed state definitions were based on symptom thresholds rather than structured diagnostic criteria and should be interpreted accordingly. Finally, although the elevated SI risk observed in hypo/mania with mixed features compared to depression with mixed features appears clinically meaningful, confidence intervals were not reported for mixed mood states due to variance instability. As a result, formal statistical comparisons with pure mood states (e.g., depression) were not possible. Despite this, the present study has several strengths including a large sample of well-characterized patients with BD and a prospective design with standardized rating scales for the clinical assessments. The broad inclusion criteria enhanced the generalizability of our findings.

## Conclusions

This study highlights individuals with hypo/mania with mixed features demonstrate an increased odd of SI, reflecting contributions from depression, hypo/mania and their interaction. These findings lay the groundwork for future research to better understand the relationship between mixed mood states and suicidality.

## Electronic supplementary material

Below is the link to the electronic supplementary material.

Supplementary Material 1

## Data Availability

No datasets were generated or analysed during the current study.

## Abbreviations

ASRM: Altman Self-Rating Mania Scale
BD: Bipolar Disorder
BD-I: Bipolar Disorder I
BD-II: Bipolar Disorder II
BD-NOS: Bipolar Disorder Not Otherwise Specified
C-SSRS: Columbia-Suicide Severity Rating Scale
CI: Confidence Interval
DSM-4: Diagnostic and Statistical Manual of Mental Disorders, Fourth Edition
DSM-5: Diagnostic and Statistical Manual of Mental Disorders, Fifth Edition
GEE: Generalized Estimating Equations
ICD-11: International Classification of Diseases, Eleventh Revision
ISBD: International Society for Bipolar Disorder
IDS-C: Inventory of Depressive Symptomatology–Clinician-Rated
IDS-C R: Inventory of Depressive Symptomatology–Clinician-Rated, Revised
IRB: Institutional Review Board
NIMH: National Institute of Mental Health
NNDC: National Network of Depression Centers
OR: Odds Ratio
PHQ-8: Patient Health Questionnaire-8
PHQ-9: Patient Health Questionnaire-9
PSR: Psychiatric Status Rating
SCID: Structured Clinical Interview for DSM Disorders
SFBN: Stanley Foundation Bipolar Network
SI: Suicidal Ideation
SPSS: Statistical Package for the Social Sciences
YMRS: Young Mania Rating Scale
