# Supplementary material for: The influence of depressive and manic symptoms on suicidal ideation in mixed mood states
Source: Int J Bipolar Disord. 2025 Jun 14;13:23. doi: 10.1186/s40345-025-00390-x (PMC12167198; doi:10.1186/s40345-025-00390-x)
Supplement: Supplementary file 1 — Supplementary Material 1 [file 40345_2025_390_MOESM1_ESM.docx]

**SUPPLEMENT OUTLINE**

eTable 1. Generalized estimating models using revised YMRS and IDS-C scales with categorical predictors

eTable 2. Full results from generalized estimating equations using full scales and categorical predictors

eTable 3. Effects of continuous scales’ scores and their interaction on suicidal ideation in generalized estimating models

**eTable 1.** Generalized estimating models using revised YMRS and IDS-C scales with categorical predictors

|  | Suicidal ideation ≥ 1 | | |
| --- | --- | --- | --- |
|  | β | 95% CI | P value ^e^ |
|  | Removing overlapping items from the YMRS | | |
| Depressive symptoms | 3.02 | 2.67 – 3.37 | 0.000 |
| Moderate hypo/manic symptoms | 1.171 | 0.28 – 2.06 | 0.01 |
| Mild hypo/manic symptoms | 0.308 | -0.14 – 0.76 | 0.18 |
| Interaction between depressive and mild hypo/manic symptoms | -0.43 | -0.95 – 0.91 | 0.105 |
| Interaction between depressive and moderate hypo/manic symptoms | -0.997 | -1.93 – -0.06 | 0.037 |
|  | Removing overlapping items from the IDS-C | | |
| Depressive symptoms | 2.974 | 2.64 – 3.31 | 0.000 |
| Moderate hypo/manic symptoms | 1.345 | 0.82 – 1.87 | 0.000 |
| Mild hypo/manic symptoms | 0.719 | 0.37 – 1.07 | 0.000 |
| Interaction between depressive and mild hypo/manic symptoms | -0.506 | -0.89 – -0.13 | 0.009 |
| Interaction between depressive and moderate hypo/manic symptoms | -0.799 | -1.37 – -0.22 | 0.006 |

*^e^* *GEE models used Bonferroni correction to adjust for multiple comparisons*

**eTable 2.** Full results from generalized estimating equations using full scales and categorical predictors

| **Variable** | | **β** | **95% CI** | **P value** ^e^ |
| --- | --- | --- | --- | --- |
| Mood states^a^ |  | | | |
|  | Depressive symptoms | 3.09 | 2.73 – 3.45 | < 0.001 |
|  | Moderate h/m symptoms | 1.135 | 0.41 – 1.87 | 0.002 |
|  | Mild h/m symptoms | 0.552 | 0.05 – 1.06 | 0.032 |
| Mood state interactions^b^ | Interaction between depressive and mild h/m symptoms | -0.43 | -0.95 – 0.91 | 0.105 |
|  | Interaction between depressive and moderate h/m symptoms | -0.843 | -1.59 – -0.1 | 0.026 |
| Mood state interactions with gender^c^ | Interaction between mixed h/m and male sex | -0.346 | -0.92 – 0.23 | 0.236 |
|  | Interaction between depressive symptoms and male sex | 0.024 | -0.27 – 0.32 | 0.871 |
|  | Interaction between moderate h/m and male sex | -1.451 | -2.58 – -0.32 | 0.012 |
|  | Interaction between euthymia and male sex | 0.113 | -0.41 – 0.64 | 0.673 |
|  | Interaction between mixed depression and male sex | -0.134 | -0.44 – 0.17 | 0.392 |
|  | Interaction between mild h/m and male sex | 0.057 | -0.61 – 0.72 | 0.866 |
| Bipolar subtype^d^ | Bipolar disorder, type II | -0.134 | -0.38 – 0.11 | 0.287 |
|  | Bipolar disorder, not otherwise specified | -0.279 | -0.99 – 0.43 | 0.443 |
|  | Schizoaffective disorder – *b*ipolar type | -0.312 | -1.01 – 0.39 | 0.383 |
| Other Predictors | Age | 0.003 | -0.01 – 0.01 | 0.613 |
|  | Week | -.001 | -.003 – 0.00 | 0.025 |

*Abbreviations: CI, confidence interval; h/m, hypomania*

*^a^ The reference category for each mood state predictor corresponds to the condition where the predictor does not fall within the range defining the specified mood state.*

*^b^ The reference category for each interaction represents conditions where the predictor does not meet the criteria defining one or both of the contributing mood states.*

*^c^* *The reference category reflects the interaction term with female sex.*

*^d^ Bipolar Disorder, Type I serves as the reference category.*

*^e^* *GEE models used Bonferroni correction to adjust for multiple comparisons*

**eTABLE 3.** Effects of continuous scales’ scores and their interaction on suicidal ideation in generalized estimating models

|  | **Suicidal ideation ≥ 1** | | |
| --- | --- | --- | --- |
|  | β | 95% CI | P value |
| Depressive symptoms | 0.133 | 0.124 – 0.142 | <0.001 |
| Hypo/manic symptoms | 0.022 | -0.005 – 0.049 | 0.115 |
| Interaction | -0.001 | -0.002 – 0.000 | 0.252 |

*Depressive symptoms: IDS-C-R scores ≥ 15; Hypo/manic symptoms: YMRS scores ≥ 12; Interaction: interaction term between IDS-C-R scores ≥ 15 and YMRS scores ≥ 12. Mixed states: total β of suicidal ideation during mixed states, derived from depressive and/or hypo/manic symptoms plus the interaction term (if significant).*
